# Determination of the temperature vs power dynamic behavior of a cryocooler via two independent methods in time and frequency domain

**DOI:** 10.1016/j.mex.2018.06.013

**Published:** 2018-06-26

**Authors:** Andrea Sosso, Paolo Durandetto

**Affiliations:** INRiM – Istituto Nazionale di Ricerca Metrologica, Torino, Italy

**Keywords:** Temperature to power dynamic response of a cryocooler with both time and frequency domain analyses, Cryocooler analysis, Transfer function, Step response

## Abstract

This report deals with the analysis of a cryocooler as a linear dynamical system around a set point, over a range of temperatures where the thermal properties can be considered constant.

The accurate knowledge of the cryocooler temperature dependence with a time dependent power stimulus allows to analyze the thermodynamical properties of the system and understand the power flow related, for example, to the cryocooler temperature fluctuations. This is useful for the design of efficient thermal dampers that are necessary for the thermal stabilization of the device under test Sosso et al. [1], Trinchera et al. [2]. Two different and independent methods for deriving the cooler dynamic (i.e. non-stationary) behavior are described using the two main approaches to mathematically represent a dynamical system: step response and transfer function.

• Using both approaches we were able to cross check results and provide an estimate of the accuracy of each method.
• The instrumentation required is typically available in physics and engineering laboratories.
• These results provide insights on cryocooler thermodynamics and design tools for cryocooler engineering.

## Graphical abstract

**Figure:**
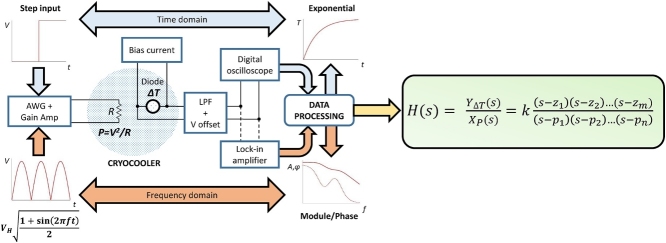

**Specifications Table**

**Table:** 

Subject area	• Engineering
More specific subject area	Cryogenics
Method name	Temperature to power dynamic response of a cryocooler with both time and frequency domain analyses
Name and reference of original method	A transfer function approach to cryocooler modeling was presented in Ref. [3], where it was estimated from the behavior of a closed-loop operated with different feedback parameters.
Resource availability	https://www.lakeshore.com/products/cryogenic-temperature-controllers/model-350/Pages/Overview.aspx https://www.tek.com/keithley-low-level-sensitive-and-specialty-instruments/keithley-ultra-sensitive-current-sources-seri http://www.thinksrs.com/products/SR560.htm https://www.keysight.com/en/pd-1000000803%3Aepsg%3Apro-pn-33250A/function-arbitrary-waveform-generator-80-mhz?cc=IT&lc=ita http://teledynelecroy.com/oscilloscope/hdo6000a-high-definition-oscilloscopes/hdo6034a http://www.ameteksi.com/products/lock-in-amplifiers/7265-dual-phase-lock-in-amplifier https://www.keysight.com/en/pd-838600-pn-6634B/100-watt-system-power-supply-100v-1a?cc=IT&lc=ita http://www.calpower.it/product.php?category_id=156&item_id=207 https://www.lakeshore.com/products/Cryogenic-Temperature-Sensors/Germanium-RTDs/Models/Pages/Overview.aspx https://www.lakeshore.com/products/cryogenic-temperature-sensors/silicon-diodes/dt-670/Pages/Overview.aspx http://www.scilab.org/

## Method details

The two methods presented here are independent and, according to the type of the measurement and to the experimental conditions [1,2], the user can choose the method that better fits his needs. Otherwise, both can be carried out in order to perform a self-consistency check, as presented in Ref. [4].

## Required equipment

The experimental setup for the correct determination of the power to temperature dynamic response of a cryocooler consists of:

- a cryogen-free cooling system, e.g. Gifford-McMahon or pulse-tube cryocooler, whose temperature to power transfer function H(s) has to be determined.
- a first calibrated low temperature sensor (e.g. a silicon diode or a germanium resistor) suitably placed in thermal contact with the cryostat for temperature monitoring and control.
- an electric heater (wounded wire or resistor) placed in thermal contact with the cold region of interest.
- a temperature controller (e.g. Lake Shore 350 or similar) for monitoring the temperature of the first sensor and raising it up to the desired operating point with a constant current (open-loop output function) through the aforementioned electric heater. If the temperature controller does not have an open-loop output option, a common dc current generator can be used.
- a 2-wire connected SMD resistor or similar with resistance of the order of 300 Ω, suitably placed over the cold region of the cryostat, necessary for supplying a certain amount of heat to the system via Joule effect; the SMD resistance value at the operating temperature point has to be known. The resistance of the two leads connecting the SMD resistor to the room temperature environment should be negligible if compared to the SMD resistance or should be precisely known and taken into account through the rest of the experiment.
- a second calibrated diode temperature sensor in thermal contact with the cold region of the cryostat for measuring its temperature variations as a result of the input power. Its voltage to temperature calibration curve has to be known and linearized over the range of interest.
- an arbitrary waveform generator (AWG) to provide the input voltage to the SMD resistor.
- a power amplifier, since normally the power provided by the AWG is not sufficient.
- a high-stability, low-noise current generator necessary for biasing the diode with an accurate dc current.
- a low-noise differential pre-amplifier to amplify the changes of diode voltage with respect to the operating point value and noise filtering.
- a dc voltage source necessary to provide the dc voltage level to be subtracted to the instantaneous diode voltage.
- a digital storage oscilloscope or similar for measuring the amplified voltage across the diode and saving data.
- a lock-in amplifier for measuring the frequency response (magnitude and phase) of the diode temperature sensor.

**Fig. 1:**
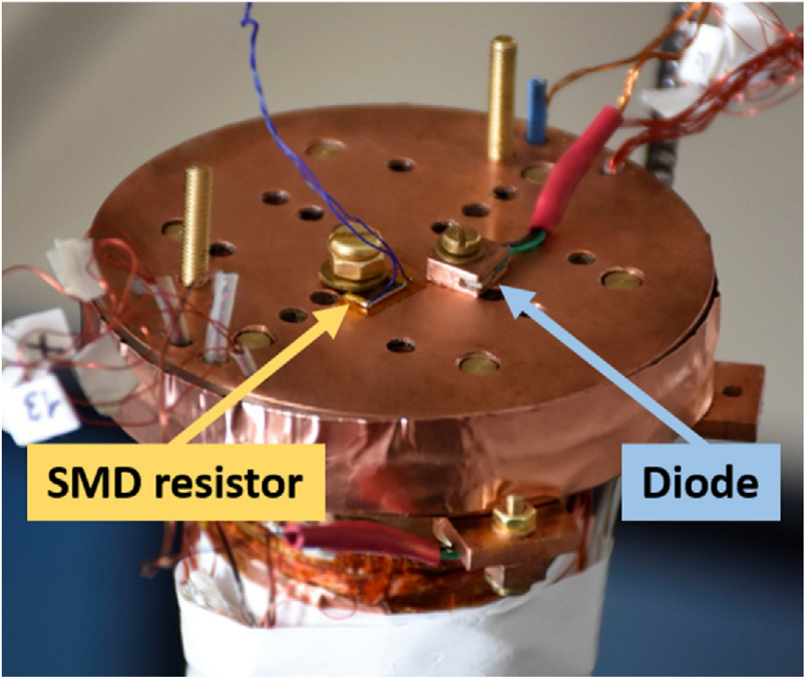
Picture of a cryocooler cold plate. A SMD resistor and a diode temperature sensor are suitably tightened to the cold plate through a brass screw. The SMD resistor is covered by a thin kapton layer to avoid accidental short-circuits.

As shown in Fig. 1, a good thermal contact between the thermal elements (resistor and temperature sensors) can be achieved by screwing all to the cold region plate (usually made of high-thermal conductivity copper). Attention has to be paid for preventing any electrical contact between the device and the cold plate that may cause undesired short-circuits.

## Measurements

In the following, the operation steps that are common for both time-domain and frequency-domain analyses are listed:

1. The cryocooler has to be set to the desired operating temperature by exploiting the open-loop heater function of the temperature controller.

2. The voltage output provided by the AWG has to be amplified by the gain amplifier

3. The diode is properly biased by an accurate dc current (10 μA for Lake Shore DT-670 [5]) provided by the current generator.

4. The dc level of the diode voltage is canceled out by subtraction of the constant compensation voltage V_DC_ (about 1.6 V at 4 K for Lake Shore DT-670 diode) provided by the dc voltage supply. This is performed by applying the diode voltage to the non-inverting input of the differential amplifier, while the dc level is applied to its inverting input. This is advantageous compared to ac signal coupling because it avoids any low frequency cutoff.

5. By setting the differential amplifier parameters, the voltage across the diode is amplified and low-pass filtered with a cut-off frequency of about 30 Hz.

### Time-domain analysis

In the time-domain analysis, a rapid power step is applied to the cryocooler. Linear system theory guarantees that the output response vs. time of transient follows a sum of exponential decays, depending on the number of poles and zeros that characterize the system.

**Fig. 2:**
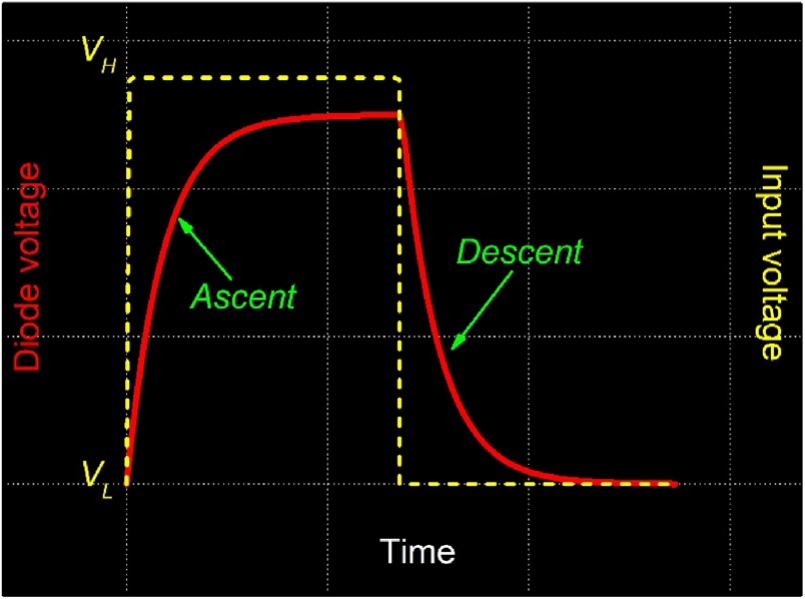
One period of the (100 s duration) step input power (yellow) and diode voltage (red) as visualized with the oscilloscope. Ascending and descending exponential decays are averaged and saved.

In the following, the subsequent operations required for the time-domain analysis are listed:

1. An adequately slow square wave voltage signal (around 10 mHz), provided by the AWG, is applied to the SMD resistor after being properly amplified by the gain amplifier. The low-level (*V*_*L*_) of the square wave is set to 0 V, while the high level (*V*_*H*_) has to be properly chosen in order to not affect the operating temperature and to maximize signal-to-noise ratio at the same time. The thermal power variation that is generated by the SMD resistor *R* in each step is given by *P = (V*_*H*_* − V*_*L*_*)*^*2*^*/R = V*_*H*_^*2*^*/R*

2. The diode voltage response is sent to the digital oscilloscope, where it is sampled, averaged and stored. The oscilloscope is set to dc coupling and high input impedance. Furthermore, oscilloscope time-scale has to be set such that at least one full period of the square wave signal is acquired, as shown in Fig. 2. The visualized diode voltage response should be an alternating sequence of exponential rise and decay profiles.

The number of averages must be chosen such that noise and cryocooler fluctuations are filtered out. Furthermore, this depends also on the required accuracy: more averages means higher accuracy, but a compromise has to be found. Indeed, an acquisition of 100 averages of one period of a 10 mHz signal would take more than 2 h.

**Fig. 3:**
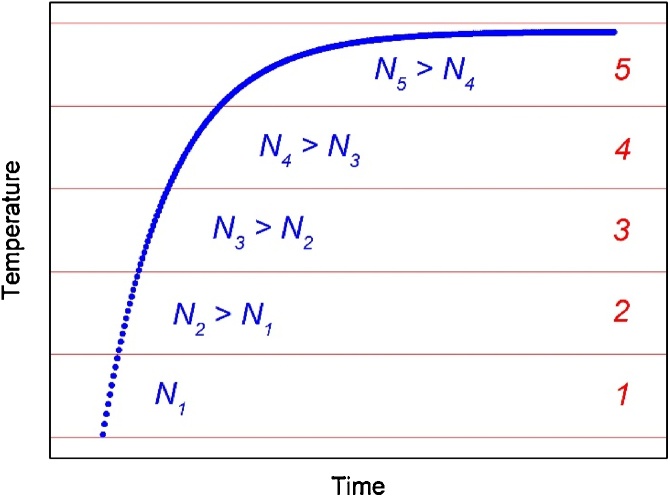
Sampled temperature-to-time response to the ascending power step input as in Fig. 1. Full temperature variation *ΔT* is subdivided into *M* = 5 equal intervals.

3. Data are collected and processed: first, each acquired voltage value has to be summed with the dc voltage V_DC_ that was previously canceled. Then, using the known calibration curve of the diode, the temperature evolution of the cold region as a result of the power excitation is obtained.

4. Data are split into ascending (from *V*_*L*_ to *V*_*H*_) and descending (from *V*_*H*_ to *V*_*L*_) parts. Each rising or descending part is separately analyzed and processed.

5. Since the oscilloscope has a constant sampling time, the number of sampled points per unit time *ΔN/Δt* is constant. This means that for high-slope temperature variations a lower number of points per interval of temperature variation *ΔN/ΔT* will be sampled, compared to lower slope deviations. This is clearly represented in Fig. 3, where the full temperature increase is subdivided into *M* equal intervals (*M* = 5 in the example), each counting a number of sampled points *N*_*i*_ that increases as the slope of the waveform under test decreases. This could affect the successive fitting procedure, since it reduces the weight of short time constants components, as a consequence of the few representative samples extracted in the first part of the transient.

**Fig. 4:**
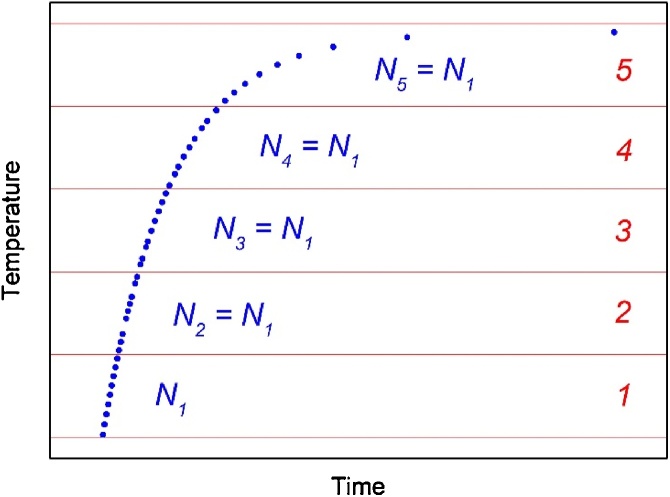
Skimmed temperature-to-time response to the ascending power step input as in Fig. 1. Full temperature variation *ΔT* is subdivided into *M* = 5 equal intervals.

Therefore, it could be useful to skim sampled data by selecting the number of points such that each temperature sub-interval will count (approximately) the same number of data points (that should coincide with the number of the less numerous interval, *N*_*1*_ in the example in Fig. 4). This data selection leads to a constant *ΔN/ΔT*.

6. Each rise or descent in temperature *ΔT* is divided by the power *P* and the result is fitted by a sum of exponential decays given by:

$$\frac{\Delta T}{P}=\sum_{i}A_{i}\left(1-e^{\frac{-t}{\tau_{i}}}\right)$$

where *A*_*i*_, *τ*_*i*_ are respectively the amplitude and the time constant of the i-th decay contribution and *ε(A*_*i*_*)* and *ε(τ*_*i*_*)* are their errors evaluated from the fit and are used in the following step.

7. A weighted average of the parameters of the two fitting functions (ascending and descending) is performed such that a unique final exponential function for *(ΔT/P)*_*avg*_ is obtained, as shown below.

$$A_{i,avg}=\frac{\frac{A_{i,asc}}{\varepsilon {\left(A_{i,asc}\right)}^{2}}+\frac{A_{i,desc}}{\varepsilon {\left(A_{i,desc}\right)}^{2}}}{\frac{1}{\varepsilon {\left(A_{i,asc}\right)}^{2}}+\frac{1}{\varepsilon {\left(A_{i,desc}\right)}^{2}}},\tau_{i,avg}=\frac{\frac{\tau_{i,asc}}{\varepsilon {\left(\tau_{i,asc}\right)}^{2}}+\frac{\tau_{i,desc}}{\varepsilon {\left(\tau_{i,desc}\right)}^{2}}}{\frac{1}{\varepsilon {\left(\tau_{i,asc}\right)}^{2}}+\frac{1}{\varepsilon {\left(\tau_{i,desc}\right)}^{2}}}$$

$${\left(\frac{\Delta T}{P}\right)}_{\mathrm{avg}}=\underset{i}{\sum }A_{i,\mathrm{avg}}\left(1-e^{\frac{-t}{\tau_{i,\mathrm{avg}}}}\right)$$

### Frequency-domain analysis

In the frequency domain analysis, electrical power with sinusoidal dependence over time is applied for a set of frequencies. Linear system theory guarantees that the output response vs. time is a sinewave, with the same frequency as the input stimulus.

In the following, the subsequent operation required for the frequency-domain analysis are listed:

1. The SMD resistor is biased at a varying voltage given by

$$V\left(t\right)=V_{H}\sqrt{\left(\frac{1+\sin\left(2\pi ft\right)}{2}\right)}$$

where *V*_*H*_ is the maximum voltage, while the low voltage *V*_*L*_ is zero. Accordingly, electrical power generated by the SMD resistor is a sinewave function with frequency *f* given by

$$P\left(t\right)=\frac{V{\left(t\right)}^{2}}{R}=\frac{{V_{H}}^{2}\left(\left[\frac{1+\sin\left(2\pi ft\right)}{2}\right]\right)}{R}$$

2. The diode voltage response is measured by the lock-in amplifier, which determines magnitude and phase of each spectral component with respect to the reference signal provided by the AWG. Lock-in time constant *τ*_*c*_ should be varied according to the reference frequency *f*, such that *τ*_*c*_* >> f*^*−1*^. The lock-in measures magnitude as the rms of the reference frequency component of the diode voltage. Linearized diode calibration curve guarantees a consistent rms voltage to temperature conversion.

**Fig. 5:**
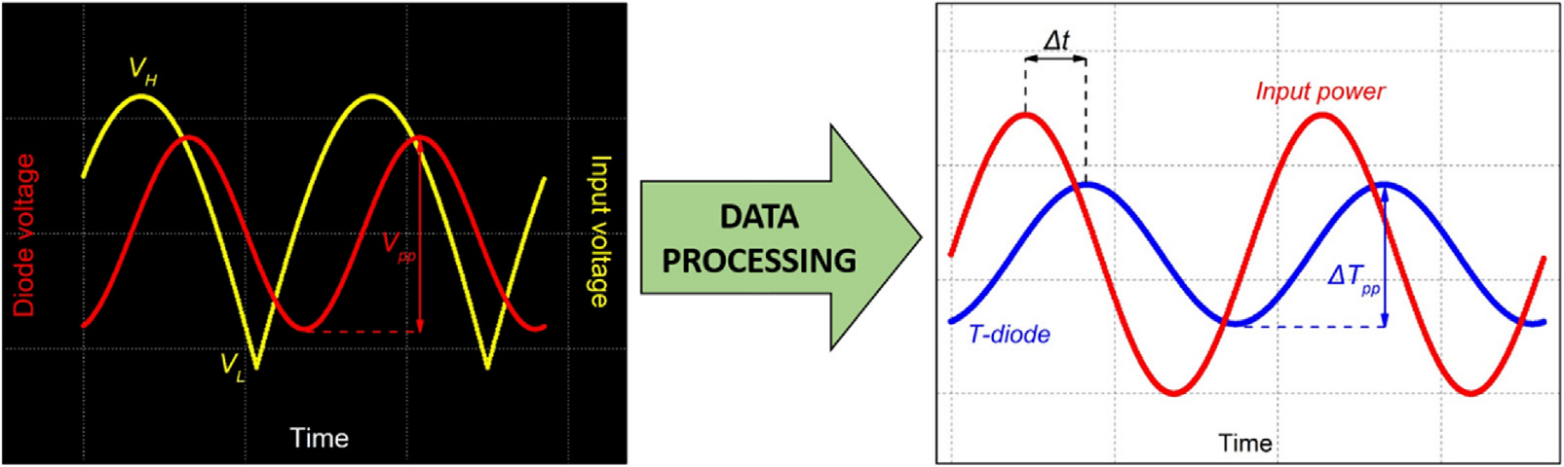
Left: diode voltage response (red) to a power sinewave over the set of frequencies in the range of interest [5] input as visualized with the oscilloscope. Sine square-root input voltage (yellow) is also measured for phase evaluation through *Δt* measurement. Right: input power sinewave (red) calculated as *V(t)*^*2*^*/R* and temperature variation (blue) measured by the diode according to its calibration curve.

If a lock-in amplifier is not available, it is possible to perform frequency-domain analysis using the digital oscilloscope, albeit with lower accuracy. In that case, the diode voltage is properly sampled and averaged as explained for the time-domain analysis. Input voltage is sampled as well, using another channel of the oscilloscope (Fig. 5). Magnitude is obtained from peak-to-peak or rms measurements and temperature conversion via the diode calibration curve. Phase *φ* can be evaluated from the delay *Δt* between the peaks of input and diode voltages as *φ = 2π∙Δt∙f*, with *φ* in radians. Nevertheless, this method is more time-consuming and less accurate compared the lock-in technique, especially when the diode voltage amplitude approaches zero. Hence, lock-in method is preferable.

3. This procedure has to be repeated for a set of frequencies in the range of interest, which usually are equally spaced in logarithmic scale.

4. Plots of magnitude and phase vs. frequency are obtained. By fitting them with a complex rational function:

$$f\left(s\right)=k\frac{\left(s-z_{1}\right)\left(s-z_{2}\right)\ldots \left(s-z_{m}\right)}{\left(s-p_{1}\right)\left(s-p_{2}\right)\ldots \left(s-p_{n}\right)}$$

it is possible to determine the number and values of the zeros (*z*_*1*_…*z*_*m*_), poles (*p*_*1*_ …*p*_*n*_) and coefficient (*k*) of the transfer function.

### Time-domain and frequency-domain analysis comparison

1. The transfer function of a system is given by the Laplace transform of its pulse response in time domain. Pulse response is simply calculable as the time derivative of step response. Therefore, we can derivate the system transfer function in two ways. First, we can differentiate in time our step response *ΔT/P*, obtaining

$$\frac{d\left(\Delta T/P\right)}{\mathrm{dt}}=\underset{i}{\sum }\frac{A_{i}}{\tau_{i}}e^{\frac{-t}{\tau_{i}}}$$

and then we can calculate its Laplace transform. The second way is to first evaluate the Laplace transform of the step response ΔT/P and then to multiply it by s, since it is known thatsT/PΔ

$$ℒ\left(\int_{0}^{t}f\left(t\right)dt\right)=\frac{ℒ\left(f\left(t\right)\right)}{s}$$

where ℒ is the Laplace transform operator and f(t) is the time-domain pulse response.f(t)

## Supplementary material

Experimental data obtained with these methods for a GM cryocooler at 4 K can be found in Ref. [6].
